# Advances in the use of nanocarriers for cancer diagnosis and treatment

**DOI:** 10.1590/S1679-45082016RB3475

**Published:** 2016

**Authors:** Débora Braga Vieira, Lionel Fernel Gamarra

**Affiliations:** 1 Hospital Israelita Albert Einstein, São Paulo, SP, Brazil.

**Keywords:** Nanomedicine, Liposomes, Nanoparticles, Neoplasms/drug therapy, Drug delivery systems

## Abstract

The use of nanocarriers as drug delivery systems for therapeutic or imaging agents can improve the pharmacological properties of commonly used compounds in cancer diagnosis and treatment. Advances in the surface engineering of nanoparticles to accommodate targeting ligands turned nanocarriers attractive candidates for future work involving targeted drug delivery. Although not targeted, several nanocarriers have been approved for clinical use and they are currently used to treat and/or diagnosis various types of cancers. Furthermore, there are several formulations, which are now in various stages of clinical trials. This review examined some approved formulations and discussed the advantages of using nanocarriers in cancer therapy.

## INTRODUCTION

**Figure 1:**
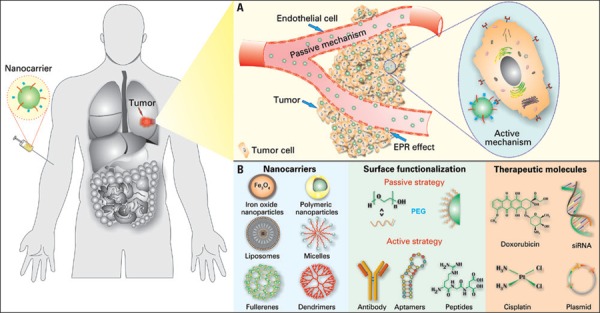
Nanocarriers for cancer treatment. (A) Nanocarriers can be accumulated in the tumor through a passive mechanism known as EPR effect, because of the increased vascular permeability in the tumor region. Additionally, the active mechanism also increase nanoparticles uptake by tumor cells. This mechanism comprises in changes on the surface of nanocarriers with molecules that can be recognized specifically by receptors on the surface of cell membrane. (B) Examples of nanocarriers surface ligands and therapeutic agents used for cancer diagnosis or treatment

EPR: enhanced permeability and retection; PEG: polyethylene glycol.

Chemotherapeutic drugs are toxic against cancer cells, but due to their low specificity and high toxicity, these drugs are also toxic for healthy cells. This toxic reaction occurs because medications, in general, are small enough molecules to pass through the endothelium in almost all regions of the organism after systematical administration, and they can reach both target regions and other regions not affected by the disease, therefore, originating a number of side effects associated with the medication. A possible strategy that may improve therapeutic efficacy of chemotherapeutic agents and decrease its side effects entails the use of colloidal nanoparticle systems. Because these drugs are encapsulated within nanoparticles of 50-800nm, they are not possible to cross the vessel wall of healthy regions of the organism (the space between these cells is only 15-30nm). This is different from what occurs in inflamed regions or even in those regions where tumors are located, in which endothelial cells are less packed among themselves than in healthy regions, which result in an accumulation of nanoparticles in the tumor tissue near blood vessel^1-3^ (Figure 1A). This vectorization strategy is known as enhanced permeability and retention effect (EPR).^4^

Surface of nanoparticles can be easily modified, allowing to direct nanocarriers to specific cancer cells with action mechanism based on expressive molecules in the surface of the tumor, which result in active directing of these particles (Figure 1A). Molecules such as antibodies, peptides and RNA aptamers, and others (Figure 1B) are widely used to direct nanoparticles.^5^ Still, the use of these nanostructure has a variety of advantages in relation to free administration of medication, and one of these advantages are: (i) protection of medication against degradation in the organism, (ii) better absorption of the drug in tumor tissue, and (iii) change in the medication’s pharmacokinetic, among others.

**Figure 2:**
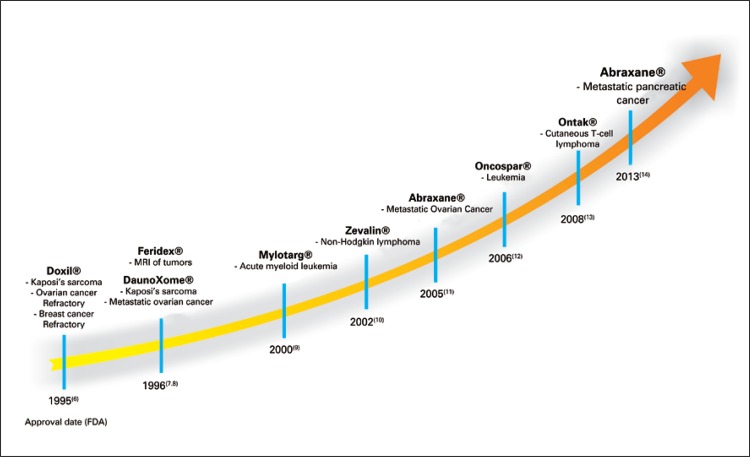
Timeline with some examples of nanoparticles approved by Food and Drug Administration.(6-14) Doxil® is the liposomal doxorubicin formulation; Feridex® includes superparamagnetic iron nanoparticles associated with dextran, DaunoXome® is the liposomal daunorubicin, Mylotarg® has gemtuzumab ozogamicin molecules bonded to monoclonal antibody, Zevalin® includes mouse monoclonal antibody IgG1 with tiuxetan chelator associated with radioactive isotope Yttrium-90, Abraxane® has paclitaxel bonded to albumin; Oncaspar® is the modified version of the L-asparaginase enzyme, and Ontak® includes the fusion protein denileukin diftitox

Currently, a number of nanoparticles have been approved by the US Food and Drug Administration (FDA)^6-14^ (Figure 2), which accumulate in solid tumors because of the EPR effect.^2,3^Among approved ones, the highlighted are liposomal doxorubicin, the Doxil^®^, which was the one of the first medication based on nanotechnology approved by FDA.^6^ Another example is the Abraxane^®^, the paclitaxel, a chemotherapy drug, which is efficiently associated with nanoparticle called albumin. This formulation was approved by FDA in 2005 for breast cancer treatment, and in 2013 to treat pancreas cancer.^11-14^ Other examples of these nanostructured medicines to treat and diagnose cancer are shown in timeline in the figure 2.

FDA: Food and Drug Administration.

Literature has several epidemiologic studies and pre-clinical tests showing the great potential of new medications either synthetic and from natural-source compounds against cancer (Table 1). These new macromolecules include peptides, proteins, oligonucleotides, plasmids and, more recently, inhibition of specific expression of gene silencing by RNA interference^3,15-17^ (Figure 1B). However, despite the great advances in science and technology to obtain new medications, pioneer pharmaceutical companies are stopping the production of new synthetic drugs to produce generic drugs, particularly after these medications patent have expired. The development of new medications is costly, particularly the synthetic routes.^3^ This scenario creates the need of presenting conventional chemotherapy drugs in new formulations which can be a new delivery system or development of new use for the existing medications, such as the case of chloroquine and their analogues, which are used for malaria treatment. And, currently, they are on trial for treatment of several types of cancer.^18^

**Table 1:** Therapeutic nanoparticles under clinical trials

Phase	Nanodrug	Type of cancer	Patients (n)	Start date	End date	Country	Study ID number
IV	Pegylated liposomal doxorubicin (Doxil^®^)	Ovary cancer	58	November 2004	January 2008	Russia	NCT00727961
I/II	Gemzar^®^ mix with compound Glycyrrhizin Injection	Pancreas cancer	60 (estimative)	May 2015	Ongoing	United States	NCT02449135
II	Cyclodextrin-containing camptothecin	Lung cancer (recurrent)	156 (estimative)	February 2013	Ongoing	United States	NCT01803269
-	Cyclodextrin-containing camptothecin	Metastatic stomach, gastroesophageal, or esophageal cancer	10	June 2012	June 2015	United States	NCT01612546
IV	Polymeric micelle containing paclitaxel	Breast cancer (recurrent)	90 (estimative)	May 2009	Ongoing	South Korea	NCT00912639
II	Magnesium oxide nanoparticles	Breast cancer	288	September 2011	August 2013	United States	NCT01439945
II	PEG-Irinotecan (NKTR 102)	Lung cancer	38 (estimative)	August 2013	Ongoing	United States	NCT01876446
I/II	Lipid nanoparticles containing siRNA	Liver cancer	72 (estimative)	December 2014	Ongoing	United States	NCT02314052
II	Block copolymer vaccine containing peptides	Melanoma	48	March 1998	November 2002	United States	NCT00003274

PEG: polyethylene glycol.

This review discusses some scientific advances related with the use of nanoparticles for cancer diagnosis and treatment. We performed the search in PubMed and Web of Science. The keywords used were “nano” AND “cancer” combined with “FDA approved” and the ‘search’ all fields. We included only articles written in English. We selected study based on number of citations and/or year of publication.

### Nanomedicines for cancer: state-of-the-art

One of the basics of nanomedicine is to delivery medications in a specific and efficient way to the site of the disease. In general, this can be achieved by different ways of administration, such as oral, nasal, transdermal, intravenous, among others. In many cases, however, the efficacy of the medication can be improved and side effects reduced by encapsulation or association to some type of nanoparticle. The main nanoparticles described in the literature are iron oxide, gold, polymeric, liposomes, micelles, fullerenes, carbon nanotubes, graphene, dendrimers, *quantum dots*, nanodiamonds, among others. Some examples of these nanocarriers are described in figure 1B.^2,3,19,20^ Next, we describe the main nanoparticles study for carrier and controlled release of medication as well as a brief report of nanocarriers current status of clinical development.

Polymeric nanoparticles are the most study particles for carrier a number of therapeutic molecules particles because of their excellent biocompatibility and biodegradability, in addition of not being toxic and non-immunogenic. They constitute a diverse class of nanocarriers because depend on the polymer that constitute them and on their surface load, they present different proprieties. Several synthetic polymers including poly (lactic acid), poly (lactic-co-glycolic acid) or polyethyleneimine, or natural, such as chitosan, collagen, gelatin or albumin, which are used to produce polymeric nanoparticles.^21,22^ One of the first studies on the use of polymeric nanoparticles for the use of cancer was reported in 1979 when Couvreur et al. developed a simple method to produce nanoparticles of poly (alkyl cyanoacrylate).^23^ Since then, nanoparticles of this polymer are intensively studied for carrier and delivery of a variety of anticancer drugs.^24^ Their study definitely contribute for the development of doxorubicin nanoparticles, which is current in phase III clinical trial.^25^ In addition, albumin nanoparticles (Abraxane^®^) have been approved by FDA for chemotherapy transportation for different types of cancer treatment.^11,14^

Amphiphilic carriers also have biologic attractive proprieties as biocompatibility, biodegradability and drug isolation of the surrounding medium, and the ability of carrier both hydrophilic and hydrophobic drugs. Liposomes, polymeric vesicles and micelles belong to the class of amphiphilic carriers (Figure 1B). Formation of liposomes results of the self-assembly of lipid molecules in aqueous solution, and they are simply closed bilayers that delimitate an internal aqueous compartment. Liposomes were the first nanocarriers approved by regulatory agencies for carrier several chemotherapeutic agents.^26,27^ As already described here, the first formulation of liposomes to be approved in the market was the Doxil^®^ in 1955 for treatment of Kaposi sarcoma associated with AIDS. Other formation of liposomes for cancer treatment are also available in the market, such as the DaunoXome^®^.^7^

Polymeric vesicles, also known as polymersomes, have a similar architecture to liposomes, since they are composed of synthetic amphiphilic polymers that have similar structure of lipids.^28^ However, we did not find any study in the literature describing clinical studies for this type of structure. Furthermore, micelles are molecular aggregates that have both hydrophilic and hydrophobic structural regions that are dynamically and spontaneously associated in aqueous solution up to a specific critical concentration. Micelles have been successful used as transporters of hydrophobic drugs.^29^ An example is the approval by FDA of the Genexol-PM for breast cancer treatment.^30^

Still, there is a number of technologies involved in the development of nanocarrier including in its chemical, physical and biological properties. For example, vectors that overcome biologic barriers, targets for cancer, releasing for the brain, combination of potential targets with antibodies with technologies and nanoparticles. However, despite several efforts towards nanocarriers, to choose the most adequate nanocarrier is not obvious for a variety of reasons that can simultaneously affect the biodistribution and target of nanocarriers. Oncologists in the near future should have specific combinations of nanocarriers and target molecules - similar to the strategies of chemotherapy combination that can be personalized to improve treatment against cancer - that will contribute to improve therapeutic results and reduce costs. These combinations will represent an important modality for cancer diagnosis and treatment.
