# Supplementary material for: Advances in the use of nanocarriers for cancer diagnosis and treatment
Source: Einstein (Sao Paulo). 2016 Jan-Mar;14(1):99–103. doi: 10.1590/S1679-45082016RB3475 (PMC4872924; doi:10.1590/S1679-45082016RB3475)
Supplement: Translation

# Translation: Avanços na utilização de nanocarreadores no tratamento e no diagnóstico de câncer

**Authors:** Débora Braga Vieira, Lionel Fernel Gamarra

**Affiliations:** 1 Hospital Israelita Albert Einstein, São Paulo, SP, Brasil.

**Keywords:** Nanomedicina, Lipossomos, Nanopartículas, Neoplasias/quimioterapia, Sistemas de liberação de medicamentos

## Abstract

A utilização de nanocarreadores como sistemas de entrega de drogas para agentes terapêuticos ou de imagem pode aumentar as propriedades farmacológicas dos compostos normalmente utilizados no tratamento e diagnóstico de câncer. Avanços em engenharia de superfície de nanopartículas para a acomodação de ligantes alvo têm feito dos nanocarreadores candidatos atrativos para um futuro trabalho envolvendo entrega de droga direcionada. Embora não direcionados, muitos nanocarreadores terapêuticos foram aprovados para uso clínico no tratamento e/ou diagnóstico de vários tipos de câncer. Além disso, há várias outras formulações que se encontram agora em estágio de testes clínicos. Este artigo de revisão examinou algumas formulações aprovadas e discutiu as vantagens da utilização de nanocarreadores na terapia de câncer.

## INTRODUÇÃO

Apesar de as drogas quimioterápicas serem tóxicas contra células cancerosas, apresentam baixa especificidade e elevada toxicidade, sendo tóxicas para células saudáveis. Isso acontece porque os fármacos, em geral, são moléculas pequenas o suficiente para atravessarem o endotélio em quase todas as regiões do organismo após administrados sistematicamente, chegando tanto às regiões alvo quanto a outras regiões que não estão relacionadas à doença, originando muito dos efeitos adversos dos medicamentos. Uma possível estratégia, que visa melhorar a eficácia terapêutica dos quimioterápicos e diminuir seus efeitos adversos, consiste na utilização de sistemas coloidais nanoparticulados. Uma vez que estes fármacos encontram-se encapsulados dentro de nanopartículas de 50 a 800nm, eles não são capazes de atravessar a parede dos vasos de regiões sadias do organismo (o espaço entre essas células é de apenas 15 a 30nm), ao contrário do que acontece em regiões inflamadas ou mesmo aquelas onde estão os tumores, nas quais células endoteliais estão menos unidas entre si que aquelas de regiões sadias, resultando num acúmulo de nanopartículas no tecido tumoral vizinho ao vaso sanguíneo^1-3^(Figura 1A). Esta estratégia de vetorização é conhecida como efeito *Enhanced Permeability and Retection (*EPR).^4^

**Figura 1:** Nanocarreadores no tratamento de câncer. (A) Nanocarreadores podem acumular no tumor por um mecanismo passivo, conhecido como efeito *Enhanced Permeability and Retection*, devido à permeabilidade vascular aumentada na região tumoral. Adicionalmente, o mecanismo ativo também aumenta a captação de nanopartículas pelas células tumorais. Esse mecanismo consiste na modificação da superfície dos nanocarreadores com moléculas que podem ser reconhecidas especificamente por receptores proteicos presentes na superfície da membrana celular. (B) Exemplos de nanocarreadores, ligantes de superfícies e agentes terapêuticos utilizados para diagnóstico ou terapia de câncer

EPR: *enhanced permeability and retection*; PEG: polietileno glicol.

Além disso, a superfície das nanopartículas pode ser facilmente alterada, de modo a direcionar o nanocarreador especificamente para células cancerosas, com mecanismo de ação baseado nas moléculas expressas na superfície celular do tumor, resultando num direcionamento ativo dessas partículas (Figura 1A). Moléculas como anticorpos, peptídeos e aptâmeros de RNA, dentre outras (Figura 1B) são as bastantes utilizadas para esse fim.^5^ Ainda, a utilização dessas nanoestruturas apresenta outras inúmeras vantagens em relação à administração da droga livre, e algumas destas vantagens são: (i) proteção do fármaco contra a degradação no organismo; (ii) melhor absorção da droga no tecido tumoral; e (iii) alteração da farmacocinética de drogas, dentre outras.

Hoje existem várias nanopartículas aprovadas pelo US *Food and Drug Administration* (FDA)^6-14^ (Figura 2), que se acumulam em tumores sólidos por conta do efeito EPR.^2,3^Dentre essas, destaca-se a formulação lipossomal de doxorrubicina, a Doxil^®^, que foi um dos primeiros medicamentos baseados em nanotecnologia aprovados pelo FDA.^6^ Outro exemplo de produto no mercado é o Abraxane^®^, no qual o quimioterápico paclitaxel encontra-se eficientemente associado à nanopartícula de albumina. Esta formulação foi aprovada pelo FDA, em 2005, para o tratamento de câncer de mama e para câncer de pâncreas, em 2013.^11-14^ Outros exemplos desses medicamentos nanoestruturados para tratamento ou diagnóstico de câncer estão mostrados na linha do tempo da figura 2.

**Figura 2:** Linha do tempo com alguns exemplos de nanopartículas de acordo com a data de aprovação clínica pelo *Food and Drug Administration*.(6-14) Doxil® é uma formulação lipossomal de doxorrubicina; Feridex® consiste em nanopartículas superparamagnéticas de ferro associadas à dextrana; DaunoXome® é uma formulação lipossomal de daunorrubicina; Mylotarg® se trata de moléculas de gentuzumabe ozogamicina ligada à um anticorpo monoclonal; Zevalin® consiste em um anticorpo monoclonal IgG1 de camundongo com o quelante tiuxetan associado ao isótopo radioativo ítrio-90; Abraxane® apresenta paclitaxel ligado à albumina; Oncaspar® é a versão modificada da enzima L-asparaginase; e Ontak® consiste em proteína fusionada chamada *denileukin diftitox*

FDA: *Food and Drug Administration*.

Existem muitos estudos epidemiológicos e testes pré-clínicos presentes na literatura, que revelam um grande potencial de novos fármacos, sejam sintéticos ou de fontes naturais, no combate ao câncer (Tabela 1). Essas novas macromoléculas incluem peptídeos, proteínas, oligonucleotídeos, plasmídeos e, mais recentemente, inibidores de expressão específica de genes por silenciamento de RNA de interferência^3,15-17^ (Figura 1B). Porém, apesar do grande avanço na ciência e na tecnologia envolvida na obtenção de novos fármacos, têm-se observado, no cenário mundial de medicamentos, que companhias farmacêuticas pioneiras têm deixado de comercializar novos fármacos sintéticos para produzirem produtos genéricos, conforme suas patentes expiram, já que o desenvolvimento de novos fármacos está relacionado aos altos custos, principalmente em rotas sintéticas.^3^ Este cenário cria a necessidade de apresentar agentes quimioterápicos clássicos sob novas formas, que podem ser um novo sistemas de entrega ou o desenvolvimento de uma nova utilização para as drogas clássicas existentes, como é o caso da cloroquina e seus análogos, que são utilizadas para tratamento de malária e, agora, estão sendo testadas no tratamento de diversos tipos de câncer.^18^

**Tabela 1:** Algumas nanopartículas terapêuticas que se encontram em ensaio clínico

Fase	Nanodroga	Tipo de câncer	Pacientes (n)	Início	Fim	País	Número de identificação do estudo
IV	Doxorrubicina lipossomal peguilada (Doxil^®^)	Ovário	58	Novembro de 2004	Janeiro 2008	Rússia	NCT00727961
I/II	Gemzar^®^ misturado com injeção de Glycyrrhizin	Pâncreas	60 (estimado)	Maio de 2015	Em andamento	Estados Unidos	NCT02449135
II	Ciclodextrina contendo camptotecina	Pulmão (recorrente)	156 (estimado)	Fevereiro de 2013	Em andamento	Estados Unidos	NCT01803269
-	Ciclodextrina contendo camptotecina	Estômago, gastresofágico ou esofágico (metastático)	10	Junho de 2012	Junho 2015	Estados Unidos	NCT01612546
IV	Micelas poliméricas contendo paclitaxel	Mama (recorrente)	90 (estimado)	Maio de 2009	Em andamento	Coreia do Sul	NCT00912639
II	Nanopartículas de óxido de magnésio	Mama	288	Setembro de 2011	Agosto 2013	Estados Unidos	NCT01439945
II	PEG-Irinotecan (NKTR 102)	Pulmão	38 (estimado)	Agosto de 2013	Em andamento	Estados Unidos	NCT01876446
I/II	Nanopartículas lipídicas contendo siRNA	Fígado	72 (estimado)	Dezembro de 2014	Em andamento	Estados Unidos	NCT02314052
II	Vacina de copolímeros em bloco contendo peptídeo	Melanoma	48	Março de 1998	Novembro 2002	Estados Unidos	NCT00003274

PEG: polietileno glicol.

O objetivo desta revisão foi discutir alguns avanços científicos relacionados ao uso de nanopartículas no diagnóstico e/ou tratamento de câncer. Para isso, bases eletrônicas, como PubMed e *Web of Science*, foram consultadas, usando as palavras-chave “*nano”ANDcancer”*, ou combinada com a palavra “FDA *approved”* no texto inteiro. A busca se limitou a artigos escritos em inglês. No caso dos artigos de revisão citados aqui, a escolha foi baseada no número de citações e/ou ano de publicação.

### Nanomedicina em câncer: o estado da arte

Um dos objetivos básicos da Nanomedicina é entregar o fármaco de forma específica e eficiente ao local da doença. Em geral, isso pode ser conseguido por diferentes vias de administração, como oral, nasal, transdérmica, endovenosa, entre outras. Em muitos casos, entretanto, a eficácia da droga pode ser melhorada, e os efeitos colaterais, reduzidos, encapsulando-o ou associando-o a algum tipo de nanopartículas. As principais nanopartículas descritas na literatura são de óxido de ferro, ouro, poliméricas, lipossomos, micelas, fulerenos, nanotubos de carbono, grafeno, dendrímeros, *quantum dots*, nanodiamantes, dentre outras*.* Alguns exemplos destes nanocarreadores estão ilustrados na figura 1B.^2,3,19,20^ A seguir, são descritas as principais nanopartículas estudadas para o carreamento e a liberação controlada de droga, bem como um breve relato de seu estágio atual de desenvolvimento clínico.

As nanopartículas poliméricas são as partículas mais estudadas para o carreamento de diversos tipos de moléculas terapêuticas, devido à sua excelente biocompatibilidade e biodegradabilidade, além de serem não tóxicas e não imunogênicas. Trata-se de uma classe diversa de nanocarreadores, porque, dependendo do polímero que as constitui e da carga em sua superfície, elas terão propriedades diferentes. Diversos polímeros sintéticos, incluindo poli (ácido lático), poli (ácido lático-co-glicólico) ou polietilenoimina, ou naturais, como quitosana, colágeno, gelatina ou albumina, são utilizados para a produção de nanopartículas poliméricas.^21,22^Um dos primeiros relatos da utilização de nanopartículas poliméricas para o tratamento de câncer foi em 1979 quando Couvreur et al. desenvolveram um método simples para a produção de nanopartículas de poli (alquil-cianoacrilato).^23^ Desde então, nanopartículas desse polímero são intensivamente estudadas para o carreamento e a liberação de diversas drogas anticancerígenas.^24^ Esse trabalho contribuiu definitivamente para o desenvolvimento de nanopartículas de doxorrubicina, atualmente em fase de estudo clínico III.^25^ Além disso, nanopartículas de albumina (Abraxane^®^) já são aprovadas pelo FDA para veiculação de quimioterápicos para o tratamento de diversos tipos de cânceres.^11,14^

Os carreadores anfifílicos também possuem propriedades biológicas atrativas, como biocompatibilidade, biodegradabilidade e isolamento de drogas do meio circundante, e a capacidade de carrear tanto drogas hidrofílicas quanto hidrofóbicas. Lipossomos, vesículas poliméricas e micelas pertencem à classe de carreadores anfifílicos (Figura 1B). A formação de lipossomos resulta da autoassociação de moléculas lipídicas em solução aquosa, e nada mais são do que bicamadas fechadas que delimitam um compartimento aquoso interno. Os lipossomos foram os primeiros nanocarreadores aprovados pelas agências reguladoras para carrear uma série de quimioterápicos.^26,27^ Como já descrito aqui, a primeira formulação lipossomal a ser introduzida no mercado foi a Doxil^®^, em 1995,^6^ para o tratamento do sarcoma de Kaposi associado à AIDS. Outras formulações lipossomais para tratamento do câncer estão também no mercado, como o DaunoXome^®^.^7^

As vesículas poliméricas, também conhecidas como polimersomos, possuem uma arquitetura semelhante à dos lipossomos, pois são constituídas de polímeros anfifílicos sintéticos, estrutura similar à dos lipídeos.^28^ No entanto, não foi encontrado nenhum artigo na literatura que descrevesse estudos clínicos para este tipo de estrutura. Já as micelas são agregados moleculares que possuem ambas as regiões estruturais hidrofílica e hidrofóbica, que, dinamicamente e espontaneamente, associam-se em solução aquosa a partir de certa concentração crítica. As micelas têm sido utilizadas com sucesso como transportadores de drogas hidrofóbicas.^29^ Por exemplo, Genexol-PM foi aprovada pelo FDA para tratamento de câncer de mama.^30^

Ainda existe uma série de tecnologias envolvidas no desenvolvimento de um nanocarreador, tanto em sua química, como sua física e sua biologia. Por exemplo, vetores que superam barreiras biológicas, alvos para câncer, liberação para o cérebro, combinação do potencial de alvos com anticorpos com tecnologia e nanopartículas. No entanto, apesar de diversos esforços direcionados na área de nanocarreadores, a escolha do nanocarreador mais adequado não é óbvia, pois vários fatores podem simultaneamente afetar a biodistribuição e o alvo dos nanocarreadores. Semelhantemente às estratégias de combinação de quimioterápicos, que podem ser personalizadas para otimizar o tratamento contra o câncer, os oncologistas, em um futuro próximo, devem contar com combinações específicas de nanocarreadores e moléculas alvo, que, certamente contribuiriam na melhora dos resultados terapêuticos e nos custos reduzidos, representando uma modalidade importante na terapêutica e de diagnóstico de câncer.
